# Strongly masked content retained in memory made accessible through repetition

**DOI:** 10.1038/s41598-021-89512-w

**Published:** 2021-05-13

**Authors:** Damian K. F. Pang, Stamatis Elntib

**Affiliations:** 1 Department of Psychological Sciences, Institute of Psychology, Health and Society, University of Liverpool, Liverpool, L69 3BX UK; 2 School of Arts and Sciences, University of Pennsylvania, Philadelphia, PA 19104 USA

**Keywords:** Consciousness, Human behaviour

## Abstract

A growing body of evidence indicates that information can be stored even in the absence of conscious awareness. Despite these findings, unconscious memory is still poorly understood with limited evidence for unconscious iconic memory storage. Here we show that strongly masked visual data can be stored and accumulate to elicit clear perception. We used a repetition method across a wide range of conditions (Experiment 1) and a more focused follow-up experiment with enhanced masking conditions (Experiment 2). Information was stored despite being masked, demonstrating that masking did not erase or overwrite memory traces but limited perception. We examined the temporal properties and found that stored information followed a gradual but rapid decay. Extraction of meaningful information was severely impaired after 300 ms, and most data was lost after 700 ms. Our findings are congruent with theories of consciousness that are based on an integration of subliminal information and support theoretical predictions based on the global workspace theory of consciousness, especially the existence of an implicit iconic memory buffer store.

## Unconscious memory

Priming effects and procedural learning are based on information being stored in the absence of conscious awareness and are among the few memory functions that remain intact in people with amnesia^1,2^. Traditional models have distinguished them from other aspects of memory under the label of implicit memory, which was seen as the only type of memory that is not contingent on conscious awareness^3,4^. The current state of research no longer supports this narrow definition of implicit memory. There are indications that subliminal stimuli may be maintained for some time and be used after a delay period to solve behavioural tasks^5–8^. Neural signatures related to memory encoding have also been described in the absence of conscious awareness^9–11^. Other studies have directly related neural data to behavioural outcomes in further support of memory encoding of subliminal information^12–15^. While the general notion of unconscious data being stored for short periods is becoming more widely accepted, the classification, function, and purpose of such memory have been controversial^16,17^. Despite a flurry of recent research, subliminal and implicit memory remain poorly understood. Here we offer evidence that repeating a strongly masked stimulus can elicit clear perception. These findings suggest that strongly masked sensory data is stored in a way that facilitates the perception of similar subsequent sensory information, becoming accessible when confirmatory evidence accumulates.

## Repetition effects

A repeated supraliminal stimulus elicits a weaker neural response than a novel one^18,19^. However, when stimuli are complex, unclear, or interrupted by noise, repetition has been found to result in a heightened neural response^20^. Repeating a supraliminal stimulus increases its bottom-up stimulus strength^21^ and improves perceptual accuracy and clarity^21–23^. Repetition effects in subliminal stimuli have not been widely studied. Early experiments with masked stimuli did not find awareness to increase through repetition^24^. However, as Atas et al. point out, this may be an artefact of the experimental design, where large inter-prime intervals introduced delays that may have suppressed repetition effects^21^. Wentura and Frings presented masked priming stimuli multiple times in quick succession. In a series of studies, they reported finding an increased priming effect without heightened prime awareness^25–28^. However, an independent research group could not replicate their findings^29^. The use of continually changing flanking letters combined with complex target words may have impaired target identification through visual crowding^30^, thus negating any gains in awareness that occurred from repetition. Gains may have been further minimised by very short presentation times (actual illumination times on the cathode-ray-tube display would have been only a fraction of the nominal values described based on the sum of refresh cycles^31^). A systematic search showed only one study by Atas et al.^21^ that provided evidence for increased awareness through repetition of priming stimuli, albeit without examining temporal effects^21^. Our study aimed to provide independent evidence on whether repetition would increase awareness as shown by Atas et al.^21^ or only impact priming but not awareness as described by Wentura and Frings^25–28^. We also wanted to examine the effects of repetition timing intervals, which has not been assessed before.

## Results

### Perceptual change

**Figure 1:**
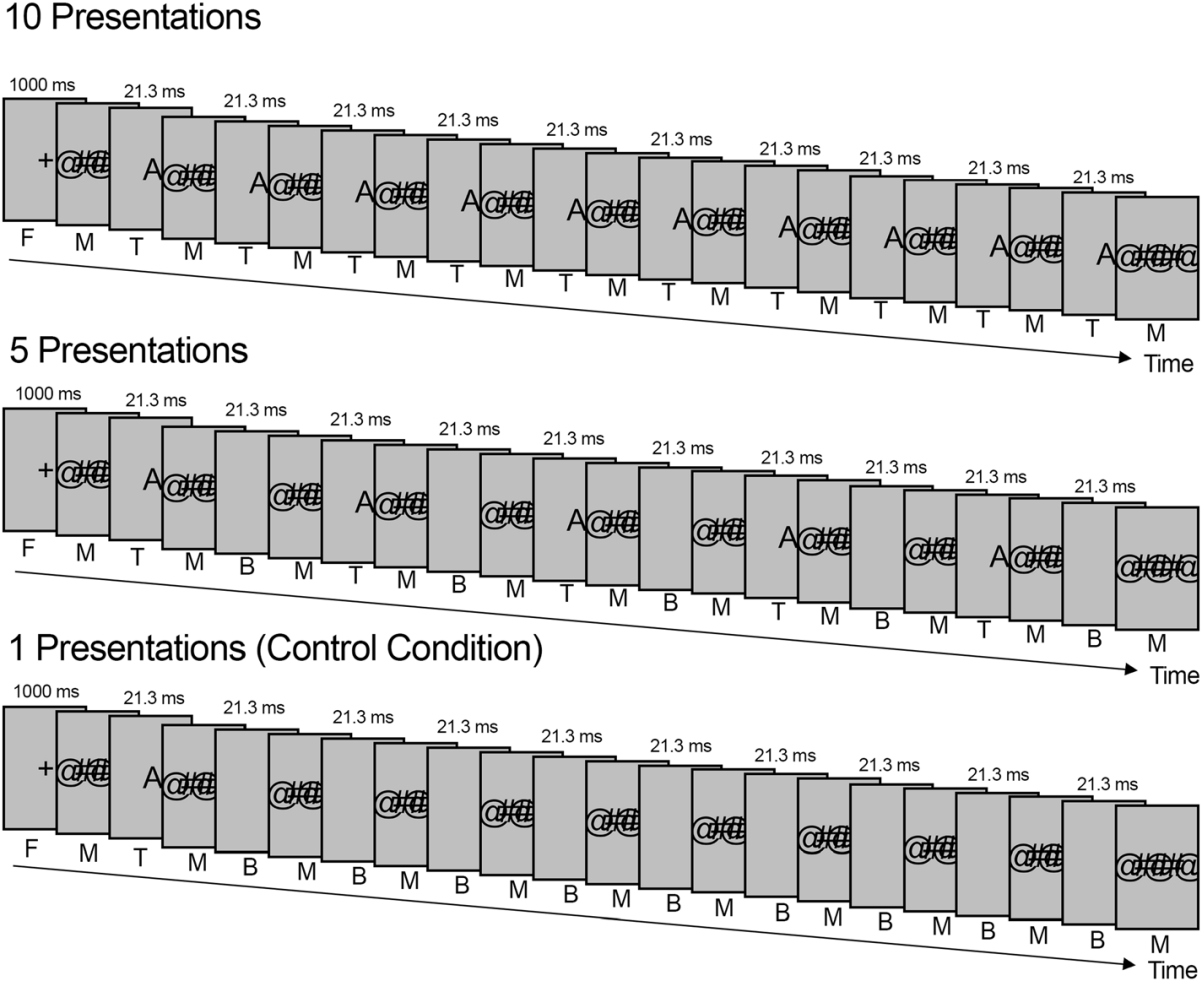
Procedure for Experiment 1. A focus signal (F) was shown followed by a series of masks (M), target letters (T), and blanks (B). The mask duration was adjusted according to target repetition-interval timings. (Experiment 2 added blank frames (7 ms) before and after the targets).

We tested the impact repetition of a strongly masked visual stimulus had on perception based on subjective and objective measures and examined associated temporal effects. In Experiment 1 we examined whether repeating a masked stimulus influenced perception across a wide range of repetition intervals (ranging from 7 ms to 8,337 ms). As shown in Fig. 1, the target stimulus was shown only once as a control condition and five or ten times in the experimental conditions. Perception was assessed through the subjective perception-awareness scale^32^ (PAS), using the adjusted PAS scale proposed by Peremen and Lamy^33^ (starting at zero to reflect the absence of experience, making the scale more intuitive). Objective performance was measured using content reports (CR) and forced-choice tasks (FCT).

**Figure 2:**
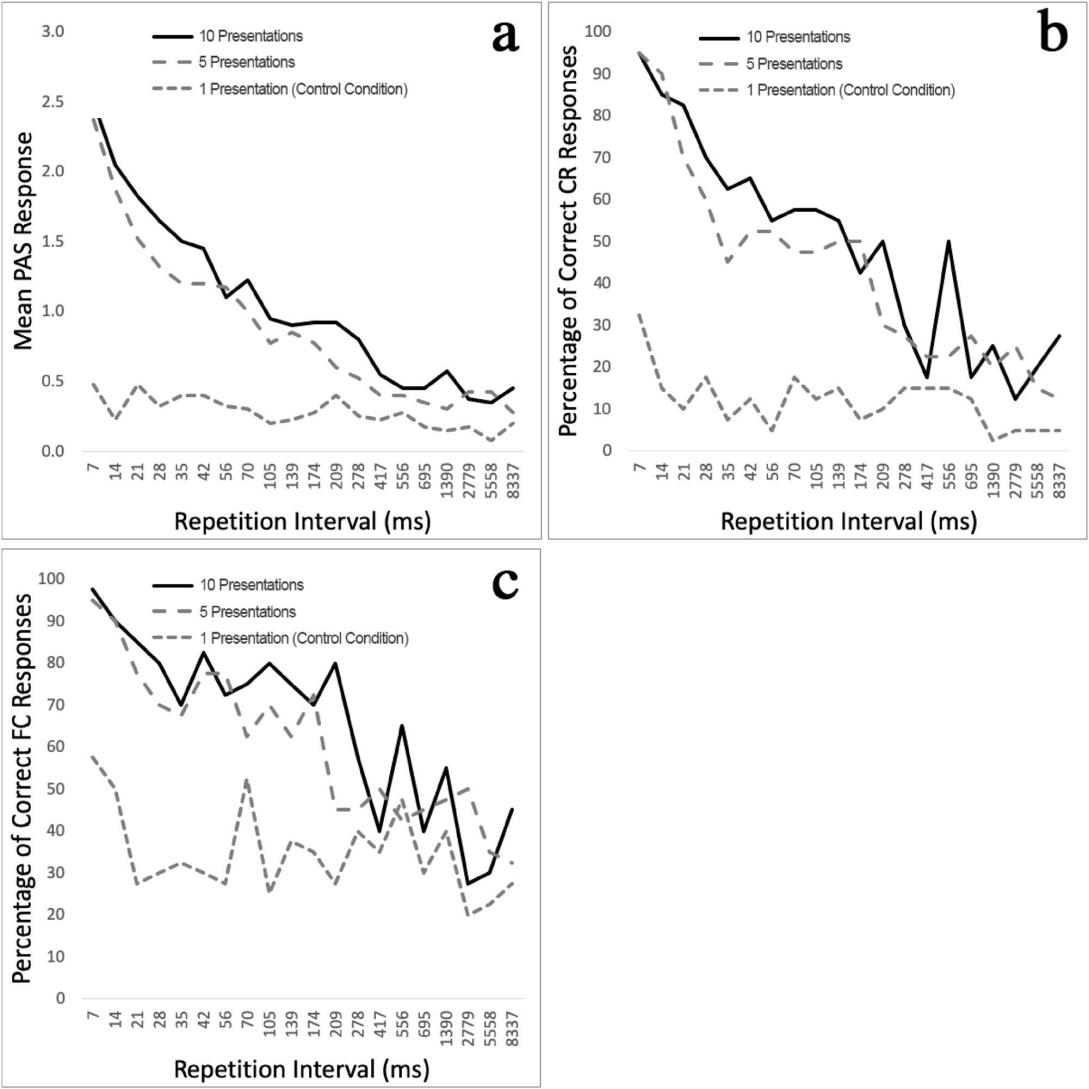
Experiment 1 results of subjective and objective measures of awareness based on the number of presentations and repetition interval timings. Results for all measures were significantly higher when masked stimuli were repeated (10 and five presentations) but decreased as repetition intervals lengthened: (**a**) Mean results of subjective perception ratings measured through the perception-awareness scale (PAS). Objective measures showed the same effects: (**b**) depicts the percentage of correct content reports (CR) while (**c**) shows the percentage of correct forced-choice task (FCT) responses.

We found that repetition significantly increased perception of masked stimuli. As expected, masking severely limited perception of target stimuli in the control condition, eliciting only minimal conscious awareness. While participants still performed above chance level in the objective measures, subjective measures showed low awareness. However, when repeated at short intervals, the same stimuli were consciously perceived despite being masked. This increased perception through repetition was significant for all measures with large effect sizes observed. Mean PAS results increased from 0.28 (*SD* = 0.50) without repetition to 0.89 (*SD* = 0.92) when shown five times and 1.05 (*SD* = 0.98) when presented ten times (Fig. 2a; detailed results are shown in Supplementary Table 1). Factorial ANOVA results showed a significant interaction effect between the number of target presentations (Factor 1) and repetition interval duration (Factor 2) for all measures—PAS *F*(10.87, 423.97) = 12.21, *p* < 0.01 (Greenhouse–Geisser correction ε = 0.29), CR *F*(14.44, 563.29) = 4.22, *p* < 0.01 (ε = 0.38), FCT *F*(15.03, 586.12) = 2.69, *p* < 0.01 (ε = 0.40). Because all measures showed some form of disordinal interactions (see Fig. 2a-c), we analysed each factor separately with a one-way repeated measures ANOVA. This procedure showed that mean PAS differences based on the number of target presentations were statistically significant *F*(1.48, 57.79) = 148.75, *p* < 0.01 η_p_^2^ = 0.79 (ε = 0.74). The same effect was observed with objective performance measures: The percentage of correct CR responses rose from 11.9% without repetition to 43.1% when presented five times and 48.9% when presented ten times (Fig. 2b; Supplementary Table 2). This effect was statistically significant, *F*(1.98, 77.25) = 149.59, *p* < 0.01, η_p_^2^ = 0.79 (ε = 0.99). FCT results increased similarly from 34.8% without repetition to 60.8% and 65.9% when presented five and ten times respectively (Fig. 2c; Supplementary Table 3) and was also statistically significant, *F*(1.93, 75.25) = 96.70, *p* < 0.01, η_p_^2^ = 0.71 (ε = 0.97).

## Temporal properties

Supraliminal visual short-term memory and iconic memory are time-sensitive and subject to temporal decay^34,35^. Implicit memory based on procedural learning has been found to persist for much longer and have been found intact for a year or more in some instances^36^. Subliminal memory is still poorly understood, and its temporal properties are largely unknown. We investigated temporal properties of repetition effects by varying the time interval between target repetitions from 7 to 8,337 ms. Perception was inversely related to repetition time intervals (Fig. 2a–c): Repetition strongly influenced perception at short intervals and waned as intervals increased. This decline is congruent with temporal decay of supraliminal short-term memory^34^. The decline was gradual and approximated levels obtained in the control condition at repetition intervals between 200 and 400 ms. The effect was mostly extinguished at repetition intervals above 700 ms. Given the significant interaction effects between the number of target presentations and repetition intervals as factors described above, we again used repeated measures one-way ANOVAs to analyse results, which were statistically significant for all measures: PAS scores *F* (8.92, 347.70) = 52.17, *p* < 0.01, η_p_^2^ = 0.57 (ε = 0.47; Supplementary Table 1), CR results *F*(12.58, 490.78) = 22.88, *p* < 0.01, η_p_^2^ = 0.37 (ε = 0.66; Supplementary Table 2), and FCT performance *F*(12.41, 483.95) = 11.34, *p* < 0.01, η_p_^2^ = 0.23 (ε = 0.65; Supplementary Table 3).

Post-hoc analysis showed that there was no significant difference between repetition intervals in the control condition for all measures (all *p* > 0.05). As such, temporal effects cannot be solely attributed to changes in masking strength.

Repetition and repetition time interval effects were captured by all measures. We used partial correlation analyses of all trials to examine the relationship between the different measures based on the number of target presentations and repetition interval durations. As shown in Supplementary Table 4, all measures were highly correlated when targets were presented 10 times and five times. These correlations in the experimental conditions were statistically significant. There was no significant correlation in the control condition without repetition.

## Experiment 2

Experiment 1 offered broad insights into a poorly understood phenomenon. Balancing this broad scope against participant fatigue^37,38^ limited the amount of data we were able to collect for each pairing of the number of target presentations and repetition interval duration, which made it difficult to filter out stochastic noise. We conducted a second experiment to address this concern. In this subsequent experiment, we also wanted to test if the general findings from Experiment 1 would hold under stronger masking conditions. Experiment 1 indicated that repetition only had a significant impact at interval lengths of less than 200 to 400 ms. We further wanted to use the second experiment to assess the impact on different interval lengths below this threshold had on perception more closely.

Experiment 2 focused on just three short intervals (35 ms, 70 ms, and 139 ms) using 10 presentations as the only experimental condition and one presentation as the control condition, since results from the previous experiment showed that results from 10 and five presentations were largely congruent (see Supplementary Tables 1–3). This reduced scope allowed us to obtain more data points from each participant for each number of target presentation-repetition interval pair without increasing the risk of participant fatigue^37^. We also increased stimulus onset asynchrony (SOA) before the onset of the mask by inserting blank frames (7 ms) to strengthen masking effects^39^ and inserted blank frames (7 ms) before each target presentation to reduce the interaction effect of forward and backward masking^40^. Apart from these changes, Experiment 2 was identical to Experiment 1.

Results from Experiment 2 supported the overall findings of Experiment 1. Mean PAS results for 10 presentations were 1.11 (*SD* = 1.00) compared to 0.40 (*SD* = 0.55) for the control condition without repetition (a detailed breakdown by intervals is shown in Supplementary Table 5). The percentage of correct CR responses (56.67%) and FCT results (68.89%) were also higher than in the control condition (CR = 15.00%; FCT = 38.33%). Two-way repeated measures ANOVA procedures showed interaction effects between the number of target presentations (Factor 1) and the repetition interval duration (Factor 2)—PAS *F*(1.76, 24.59) = 29.06, *p* < 0.01 (Greenhouse–Geisser correction ε = 0.88), CR *F*(1.57, 21.97) = 12.07, *p* < 0.01 (ε = 0.78), FCT *F*(1.52, 21.26) = 9.47, *p* < 0.01 (ε = 0.76). To understand the main effects better, we analysed the differences between the number of target presentations using repeated-measure two-tailed *t*-tests and the repetition intervals through one-way repeated measures ANOVAs. There were statistically significant differences between trials with and without repetition with large effect sizes: PAS results *t*(14) = 6.21, *p* < 0.01 *d* = 1.37, percentage of correct CR responses *t*(14) = 9.60, *p* < 0.01 *d* = 2.32, and percentage of correct FCT results *t*(14) = 8.37, *p* < 0.01 *d* = 1.60.

**Figure 3:**
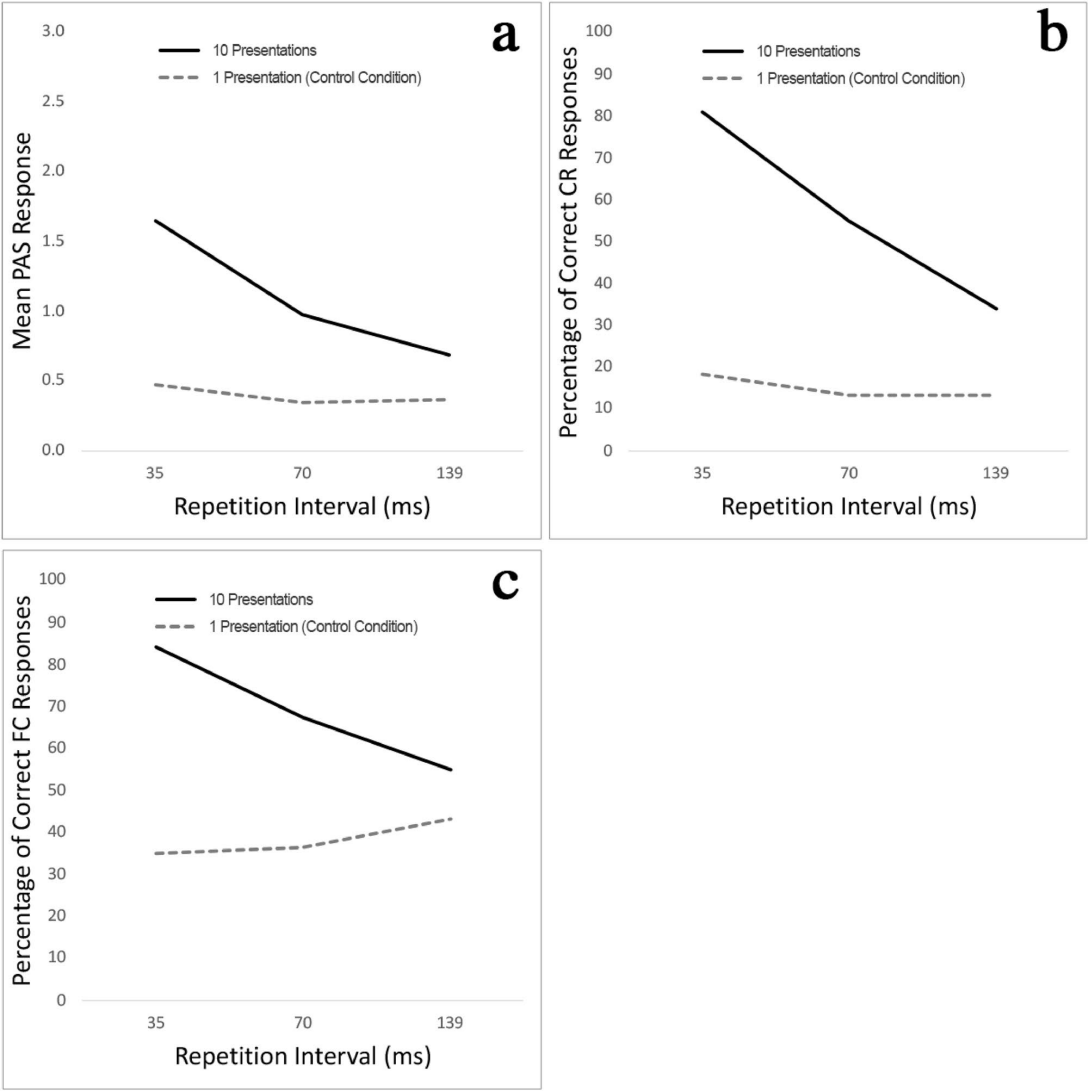
Experiment 2 results. More data points for each number of target presentation and repetition interval pair and stronger masking corroborated the findings from Experiment 1: Repetition had a significant impact on PAS reports (**a**), as well as the percentage of correct CR (**b**) and FCT responses (**c**). Repetition interval length had a significant impact on perception even among relatively short intervals.

As shown in Fig. 3, results for the experimental condition were again inversely related to the repetition interval length. Repetition intervals had significant impact on all measures when repeated: PAS *F*(1.93, 27.01) = 40.47, *p* < 0.01 η_p_^2^ = 0.74 (ε = 0.96); CR *F*(1.63, 22.81) = 19.57, *p* < 0.01 η^2^_p_ = 0.58 (ε = 0.81), and FCT *F*(1.40,19.65) = 11.46, *p* < 0.01 η_p_^2^ = 0.45 (ε = 0.70). Post-hoc tests indicated no statistically significant difference for any pairwise comparison in the control condition (dashed slopes in Fig. 3a-c).

## Discussion

Our findings show that strongly masked stimuli can elicit clear perception through repetition. While one-way analyses showed that both the number of target presentations and the repetition intervals had significant impacts on perception on their own, factorial analyses showed significant interaction effects between the number of target presentations and repetition intervals. The combination of repeating a stimulus and short intervals strongly improved perception while other combinations (short intervals without repetition or repetition with long intervals) resulted in poor perception (see Supplementary Data Tables 1–3 and 5). Our results support the idea that repetition of masked stimuli improves perception, as described by Atas et al.^21^ and diverge from the results of Wentura and Frings^25–28^. As described in the introduction, this divergence may be due to visual crowding^30^ and timing issues in their studies^31^.

## Levels of awareness

Performance in the control condition was above chance levels for objective measure tasks. As such, it is not clear whether masking rendered target stimuli completely unconscious as is indicated by subjective awareness ratings. Objective measures, like forced-choice tasks, are highly sensitive but suffer from poor exclusiveness: They are influenced by subliminal processes, such as priming effects, and thus, may capture some unconscious content^41^. Kouider and Dehaene offer converging evidence of how unconscious stimuli can still be processed extensively by the brain resulting in above chance performance in some objective tasks^42^, which could explain the divergence between subjective and objective results in our study. Others have described this as unconscious guessing similar to blindsight^43^. This may have been amplified by using single letters rather than more complex stimuli like words. While it is possible that above chance performance in our study was due to priming effects rather than based on conscious perception, the only evidence for this comes from subjective reports, which may suffer from biases. For this reason, the level of awareness without repetition needs to be interpreted with caution. We can, however, confidently state that masked stimuli in the control condition were poorly perceived and that repetition dramatically improved perception on both subjective and objective measures.

## Explanatory frameworks

Repetition increases the number of detection opportunities. Similarly, if we assume that each masked presentation had an above zero probability of being detected, then increased perception through repetition could simply be the result of an increase in the overall probability of the masked stimulus being detected. Atas et al. described this as a *probability summation*^21^. Our data indicates that neither was the case: Experiment 1 showed that repetition had little effect on perception during long intervals, where results were comparable to the control condition despite having the same amount of detection opportunities and probability summation (see Supplementary Tables 1–3). Our results show no statistically significant difference in perception between different timing intervals in the control condition. As such, differences across repetition intervals cannot be solely attributed to differences in masking duration. Trials with five presentations in Experiment 1 offered half as many detection opportunities as trials with 10 presentations but resulted in similar levels of perception, further indicating a categorical change in perception rather than a result solely reflecting detection chances. Neither the number of detection opportunities nor probability summation can account for the temporal properties observed in our experiments.

The impact of timing intervals renders explanations based on the summation of individual elements very unlikely, regardless of which property of an individual element is invoked. Likely explanations have to take interactions between individual presentations into account. Any form of interaction between target stimuli requires some information to be retained. Atas et al. suggest that this may be achieved through *physiological summation*^21^, which is based on the idea that individual masked stimuli briefly activate higher processing areas and that reactivation within a short time frame through repetition can activate the horizontal connections of these areas. This activation triggers *recurrent processing* by sending top-down signals that amplify incoming bottom-up information^21,44,45^. This model suggests that early presentations create short-term changes in the neural pathways that change perception of subsequent stimuli, akin to a form of short-term neural sensitization^46^. In essence, physiological summation suggests that some information is briefly retained in the form of altered states of neural networks. Although this form of memory facilitates the processing of subsequent stimuli, it does not in itself contain accessible content. This presents a plausible explanation for our findings but also raises several questions. Once recurrent processing is fully ignited it supresses closely following bottom-up inputs (or *feedforward sweeps*)^44^. Both the mask and the target were repeated in our experiment. According to recurrent processing theory, one signal would block the other out. It is not clear why the repeated target would be given priority over the repeated mask, especially when considering that sequences started and ended with a mask (see Fig. 1). During intermediate intervals masking stimuli were shown for much longer durations than target stimuli, making it much more likely for the mask rather than the target to ignite recurrent processing. It is also unclear how physiological summation can account for the gradual decline in perception as interval timings increased. It is possible that recurrent processing only occurred at lower levels at these intervals or that it was only partially ignited. While possible, these explanations seem contrived and do not clearly follow from the model itself. They also do not address how this results in an intermediate form of awareness and why the masks with their greater bottom-up strength did not supress these processes. While our findings cannot discount physiological summation as an underlying mechanism, it seems unsatisfactory as an overall explanation.

Another possible explanation comes from the *global workspace theory* (GWT) of consciousness^47^ and its various offshoots^42,48^, which describe conscious awareness as the result of integrating unconscious processes^42,48^. Within this framework, perception occurs as a result of an accumulation of subliminal evidence combining top-down and bottom-up processes^42^. For accumulation to occur, incoming sensory data needs to be stored. Kouider and Dehaene predicted the existence of a non-conscious buffer store that retains information for at least a few hundreds of milliseconds^42^. Elements of this buffer store enter awareness when selective attention is directed towards them^42,48,49^. An extension of this theory suggests that this buffer store can be accessed at different hierarchical levels, resulting in the possibility of partial awareness of stored content^50^. The fact that different levels can be extracted implies that the raw data stored is precategorical, which, when combined with the predicted short duration would make it akin to a form of implicit iconic memory. However, there is currently no direct evidence for such a memory store (see^43^).

Our study solely examined behavioural responses to repeated masked stimuli. As such, we cannot directly evaluate the mechanisms that underlie our findings, leaving room for different explanations. But there is strong evidence that some information of the target stimulus was retained for short periods of time despite being masked. Our results also indicate that this retention is time sensitive and showing a gradual decline with meaningful extraction becoming severely compromised after around 300 ms and almost completely lost around 700 ms. This time course closely matches the temporal properties of iconic memory^34^. Iconic memory has been described as precategorical, highly detailed, and being eliminated by new stimuli^51^. Our study did not examine whether retained information was categorical or if a non-categorical scene was analysed and interpreted during responses. Similarly, we did not directly assess the level of detail. However, our results indicate that retained information fades gradually rather than suddenly in an all-or-none fashion, which makes it less likely to be categorical given that target stimuli were single letters. While the time course of this retention conforms to iconic memory, it diverges from this type of memory in two other crucial aspects: Firstly, unlike iconic memory, subjective reports suggest that participants had very little awareness of the stored information without repetition. Similarly, although objective measure performance was above chance, it was still poor in the control condition and very unlike what would be expected if it was stored in iconic memory. Secondly, this information was not eliminated by new stimuli (i.e. the mask in our study).

Our data fits well with the theoretical predications of Kouider and Dehaene^42^. The study of Atas et al.^21^ likewise provides supporting evidence, especially when combined with our findings on temporal sensitivity that make alternative explanations less likely. Above chance performance in objective measures in our study and comparatively higher PAS values in the control condition in the study of Atas et al. (PAS = 1.9, or 0.9 with the Peremen and Lamy adjustment^33^ used here) raise some question on whether stored information was fully unconscious. This does not render these results incompatible with Kouder and Dehaene’s prediction, especially when including the partial awareness hypothesis. The prediction of hierarchical access provides a convincing explanatory framework for how information from a gradually decaying memory store can result in the partial awareness we observed. While we cannot exclude other explanations that would predict the same outcome, the predicted memory buffer as part of GWT seems to be a perfect fit both our data and those of Atas et al.^21^. Conversely, these two sets of data provide empirical support for this theory and are, to our knowledge, the first direct empirical evidence fur such a memory buffer and the first to describe its time course and decay properties.

## Visual masking

Visual masking effects have been extensively studied for over a century, but the underlying mechanisms are still poorly understood^39,52^. Traditional models have described visual masking as rendering stimuli invisible by erasing their memory representations^53^. Although this erasure hypothesis has been questioned^54–56^ direct evidence against it has been elusive, and the hypothesis is still the dominant explanation for visual masking effects^39,48,57^. According to this erasure hypothesis, repetition of subliminal stimuli would have a negligible impact on perception because memory traces from each presentation would be removed prior to the onset of the repeated iteration. Our findings suggest that visual masking does not erase memory representations of preceding stimuli but rather limits conscious awareness of them. Our finding that conscious awareness could be elicited through repetition suggests that subliminal and supraliminal memory are not categorically incompatible but dynamically related. The combination of visual masking and repetition described here offers a way to control perception of stimuli with a high level of precision and may be a useful method for future research in the areas of perception, consciousness, memory, and attention.

## Method

### Ethics statement

The University of Liverpool issued ethics and review board approval for this study. All methods were performed in accordance with the relevant guidelines and regulations. Participation was voluntary without any rewards offered and kept confidential. Written informed consent was obtained from all participants after providing all relevant information and giving an opportunity to ask any questions. Data was coded anonymously.

### Participants

Recruitment advertisements were placed on social media platforms. Experiment 1 comprised 40 participants (16 male, 24 female) aged between 18 and 66 (*M* = 38.5, *SD* = 13.08). Experiment 2 was based on 15 participants (7 male, 8 female) aged between 20 and 61 (*M* = 34.6, *SD* = 12.96). All participants had normal or corrected-to-normal vision.

### Apparatus and materials

Stimuli were presented on a 144 Hz BenQ XL2411 liquid–crystal display (LCD) and controlled by the software DMDX 5.1.4.2. Recent advancements in technology render earlier criticisms of slow response times for LCDs invalid^58^ while poor spatial consistency only occurs at obtuse viewing angles and was of no concern for the present study where stimuli were presented centrally in straight line of sight in a pre-cued location^59^. The sample-and-hold technology of LCDs allows for continuous illumination of stimuli during the presentation period, which offers increased ecological validity compared to the pulsed presentation of other display technologies^60–62^. Stimulus timing errors and misspecifications can undermine the validity of results and prevent replicability^31,63^. We extensively tested our apparatus for stimulus presentation accuracy using a photodiode and oscilloscope to avoid this problem. Supplementary Fig. 3 shows an averaged time course of a stimulus presentation, highlighting the rise time (the time illumination took to reach from 10 to 90% of the target), the fall time (time for illumination to fall below 10% from 90%), the overdrive peak, and the continuous presentation. Supplementary Table 6 shows average stimulus presentation timings and 95% confidence intervals based on test results, which include apparatus latency and display response delays. They include the rise and fall times shown in Supplementary Fig. 3.

Target stimuli were upper case letters (font Arial) presented in black colour at the centre of the screen on a grey background (kilo colour code 192,192,192) presented in font size 30, corresponding to a visual angle of 1.0º in height at a viewing distance of 70 cm. Masks consisted of superimposed hash (“###”) and at (“@@@”) symbols.

### Experiment 1 procedure

Experiment 1 followed a repeated-measures paradigm, where each participant was tested on all conditions. The experiment was conducted in a brightly lit room (approximately 1,300 lx) with the LCD monitor set to full brightness (rated at 350 cd/m^2^). The experiment was self-paced: Participants started each trial by pressing the spacebar when they were ready. A plus sign (“ + ”) was shown as a focus signal for 1,000 ms, followed by a series of masks interspersed with the target stimulus or blank frames (Fig. 1). A randomly chosen target letter (drawn from the 26 letters in the English alphabet) was presented for 21.3 ms, (95% CI [21.28, 21.36]) and was shown ten times, five times or only once as a control condition. Each of these conditions was tested using 20 different repetition intervals (with blank screens used to reduce the number of repetitions when targets were presented five times or only once) resulting in a total of 60 trials per participant. Trial order was randomised and unique for each participant.

After each trial, perception was evaluated using three measures in successive order: First, participants were asked what letter they saw between the masks and write their answer in a blank field as a content report (CR). Participants were asked to guess if unsure. In a second step, subjective perception was measured using the four-point perception-awareness scale (PAS)^32^ incorporating Peremen and Lamy’s recommended scale adjustment of starting at zero to reflect the absence of experience to make the scale more intuitive^33^. Participants were asked how clear the experience was. Answer options based on the adjusted PAS were “0 = No experience”, “1 = Weak glimpse”, “2 = Almost clear image”, “3 = Clear image.” The third and final measure comprised completing a forced-choice task (FCT): Participants were asked which letter was shown and were given four answer options in a randomised order that included the correct answer and three randomly generated alternatives drawn from the same pool as the target letters. As such, participants were likely to encounter incorrect answer options as target letters in other trials. Participants were again asked to guess if unsure. No feedback was provided during the experiment other than a progress report after completing 25%, 50%, 75% and 100% of trials.

Consciousness is ultimately a phenomenological occurrence. hence the use of PAS as a primary measure. We used CR because they allow an objective evaluation of this subjective experience and because they capture a content-awareness dimension that may be missing from the PAS^64^. However, CRs fare poorly on exhaustiveness: They fail to capture some conscious content^41^. FCTs were used to fill this gap. FCTs have been shown to be highly exhaustive but suffer from poor exclusiveness^41^: They are influenced by subliminal processes, such as priming effects, and capture content that may not be conscious. Our research is exploring a novel phenomenon. The combination of measures used allowed us to capture different dimensions of this effect and to test whether it is robust across these dimensions. There is a possibility that using multiple measures results in earlier assessments cueing later responses. In our study, CR responses may prompt FCT choices. Since choosing from given options is an easier task than making a report without any cues, trials with correct CR responses would likely result in correct FCT answers without cueing. Each FCT had only three incorrect options. Thus, an incorrect CR response would only be presented as an option in a few cases. Nonetheless, FCT results need to be interpreted with caution due to the possibility of cueing.

## Experiment 2

Experiment 2 was identical to Experiment 1 except for the following changes: Experiment 2 only tested 10 presentations as an experimental condition and one presentation as the control condition at intervals of 35 ms, 70 ms, and 139 ms. Blank frames lasting 7 ms were inserted before and after the target to increase masking strength^39,40^. Each participant completed 48 trials.

Data were analysed using IBM SPSS Statistics 21, RStudio 1.2.1335, and Python 3.7.6 (using the NumPy, Pandas, Matplotlib, and Pingouin packages). Repeated measures analyses of variance (ANOVA) and repeated measures *t* tests were used to test the main hypotheses while partial correlations were run to examine the relationship between measures.

## Supplementary Information

Supplementary Information.

## Data Availability

The data that support the findings of this study are available from the corresponding author upon reasonable request.
