# Supplementary material for: Strongly masked content retained in memory made accessible through repetition
Source: Sci Rep. 2021 May 13;11:10284. doi: 10.1038/s41598-021-89512-w (PMC8119432; doi:10.1038/s41598-021-89512-w)
Supplement: Supplementary file 1 — Supplementary Information. [file 41598_2021_89512_MOESM1_ESM.pdf]

Strongly masked content retained in memory made accessible through repetition

Damian K. F. Pang & Stamatis Elntib

Supplementary Information

Supplementary Information

Supplementary Data Table 1 | Detailed perception-awareness scale (PAS) results of Experiment 1.

Repetition Interval (ms)	10 Presentations		5 Presentations		1 Presentation (Control Condition)	
7.4	2.50	[2.24, 2.76]	2.38	[2.12, 2.63]	0.48	[0.27, 0.68]
14.4	2.05	[1.73, 2.37]	1.88	[1.57, 2.18]	0.23	[0.09, 0.36]
21.3	1.83	[1.52, 2.13]	1.53	[1.22, 1.83]	0.49	[0.31, 0.67]
28.3	1.65	[1.34, 1.96]	1.33	[1.04, 1.61]	0.33	[0.16, 0.49]
35.2	1.50	[1.18, 1.82]	1.20	[0.90, 1.50]	0.40	[0.19, 0.61]
42.2	1.45	[1.15, 1.75]	1.20	[0.91, 1.49]	0.40	[0.23, 0.57]
56.1	1.10	[0.83, 1.37]	1.18	[0.90, 1.45]	0.33	[0.17, 0.48]
70.0	1.23	[0.97, 1.48]	1.00	[0.78, 1.22]	0.30	[0.13, 0.47]
104.7	0.95	[0.69, 1.21]	0.78	[0.54, 1.01]	0.20	[0.07, 0.33]
139.4	0.90	[0.64, 1.16]	0.85	[0.61, 1.09]	0.23	[0.09, 0.36]
174.2	0.93	[0.68, 1.17]	0.78	[0.56, 0.99]	0.28	[0.11, 0.44]
208.9	0.95	[0.69, 1.21]	0.60	[0.37, 0.83]	0.40	[0.20, 0.60]
278.4	0.80	[0.58, 1.02]	0.53	[0.33, 0.72]	0.25	[0.09, 0.41]
417.3	0.55	[0.35, 0.75]	0.40	[0.23, 0.57]	0.23	[0.07, 0.38]
556.2	0.45	[0.26, 0.64]	0.40	[0.24, 0.56]	0.28	[0.13, 0.42]
695.2	0.45	[0.27, 0.63]	0.35	[0.18, 0.52]	0.18	[0.05, 0.30]
1389.9	0.58	[0.34, 0.81]	0.31	[0.16, 0.46]	0.15	[0.03, 0.27]
2779.3	0.38	[0.20, 0.55]	0.43	[0.21, 0.64]	0.18	[0.03, 0.32]
5558.1	0.35	[0.20, 0.50]	0.43	[0.22, 0.63]	0.08	[-0.01, 0.16]
8336.9	0.45	[0.25, 0.65]	0.28	[0.07, 0.48]	0.20	[0.02, 0.38]

Mean PAS results for each condition and repetition-interval pair with calculated 95% confidence intervals shown in brackets.

Supplementary Data Table 2 | Detailed content report (CR) results of Experiment 1.

Repetition Interval (ms)	10 Presentations		5 Presentations		1 Presentation (Control Condition)	
7.4	95.0	[87.5, 100.0]	95.0	[87.5, 100.0]	32.5	[20.0, 47.5]
14.4	85.0	[72.5, 95.0]	90.0	[80.0, 97.5]	15.0	[5.0, 27.5]
21.3	82.5	[70.0, 92.5]	70.0	[55.0, 85.0]	10.0	[2.5, 20.0]
28.3	70.0	[55.0, 82.5]	60.0	[45.0, 75.0]	17.5	[7.5, 30.0]
35.2	62.5	[47.5, 77.5]	45.0	[30.0, 60.0]	7.5	[0.0, 15.0]
42.2	65.0	[50.0, 80.0]	52.5	[37.5, 67.5]	12.5	[2.5, 22.5]
56.1	55.0	[40.0, 70.0]	52.5	[37.5, 67.5]	5.0	[0.0, 12.5]
70.0	57.5	[42.5, 72.5]	47.5	[32.5, 62.5]	17.5	[7.5, 30.0]
104.7	57.5	[42.5, 72.5]	47.5	[32.5, 62.5]	12.5	[2.5, 22.5]
139.4	55.0	[40.0, 70.0]	50.0	[35.0, 65.0]	15.0	[5.0, 27.5]
174.2	42.5	[27.5, 57.5]	50.0	[35.0, 65.0]	7.5	[0.0, 17.5]
208.9	50.0	[35.0, 65.0]	30.0	[15.0, 45.0]	10.0	[2.5, 20.0]
278.4	30.0	[17.5, 45.0]	27.5	[15.0, 42.5]	15.0	[5.0, 27.5]
417.3	17.5	[7.5, 30.0]	22.5	[10.0, 35.0]	15.0	[5.0, 27.5]
556.2	50.0	[35.0, 65.0]	22.5	[10.0, 35.0]	15.0	[5.0, 27.5]
695.2	17.5	[7.5, 30.0]	27.5	[15.0, 42.5]	12.5	[2.5, 22.5]
1389.9	25.0	[12.5, 37.5]	20.0	[7.5, 32.5]	2.5	[0.0, 7.5]
2779.3	12.5	[2.5, 22.5]	25.0	[12.5, 37.5]	5.0	[0.0, 12.5]
5558.1	20.0	[7.5, 32.5]	15.0	[5.0, 27.5]	5.0	[0.0, 12.5]
8336.9	27.5	[15.0, 42.5]	12.5	[2.5, 22.5]	5.0	[0.0, 12.5]

Percentage of correct CR results for each condition and repetition-interval pair with 95% CI in brackets (obtained through a bootstrap procedure using 5,000 samples).

Supplementary Data Table 3 | Detailed forced-choice task (FCT) results of Experiment 1.

Repetition Interval (ms)	10 Presentations		5 Presentations		1 Presentation (Control Condition)	
7.4	97.5	[92.5, 100.0]	95.0	[87.5, 100.0]	57.5	[42.5, 72.5]
14.4	90.0	[80.0, 97.5]	90.0	[80.0, 97.5]	50.0	[35.0, 65.0]
21.3	85.0	[72.5, 95.0]	77.5	[65.0, 90.0]	27.5	[15.0, 42.5]
28.3	80.0	[67.5, 92.5]	70.0	[55.0, 85.0]	30.0	[15.0, 45.0]
35.2	70.0	[55.0, 85.0]	67.5	[52.5, 82.4]	32.5	[17.5, 47.5]
42.2	82.5	[70.0, 92.5]	77.5	[65.0, 90.0]	30.0	[17.5, 45.0]
56.1	72.5	[57.5, 85.0]	77.5	[65.0, 90.0]	27.5	[15.0, 42.5]
70.0	75.0	[60.0, 87.5]	62.5	[47.5, 77.5]	52.5	[37.5, 67.5]
104.7	80.0	[67.5, 90.0]	70.0	[55.0, 82.5]	25.0	[12.5, 37.5]
139.4	75.0	[60.0, 87.5]	62.5	[47.5, 77.5]	37.5	[22.5, 52.5]
174.2	70.0	[55.0, 82.5]	72.5	[57.5, 85.0]	35.0	[20.0, 50.0]
208.9	80.0	[67.5, 92.5]	45.0	[30.0, 60.0]	27.5	[15.0, 42.5]
278.4	57.5	[40.0, 72.5]	45.0	[30.0, 60.0]	40.0	[25.0, 55.0]
417.3	40.0	[25.0, 55.0]	50.0	[35.0, 65.0]	35.0	[20.0, 50.0]
556.2	65.0	[50.0, 80.0]	42.5	[27.5, 57.5]	47.5	[32.5, 62.5]
695.2	40.0	[25.0, 55.0]	45.0	[30.0, 60.0]	30.0	[15.0, 42.5]
1389.9	55.0	[40.0, 70.0]	47.5	[32.5, 62.5]	40.0	[25.0, 55.0]
2779.3	27.5	[15.0, 42.5]	50.0	[35.0, 65.0]	20.0	[7.5, 32.5]
5558.1	30.0	[15.0, 45.0]	35.0	[20.0, 50.0]	22.5	[10.0, 35.0]
8336.9	45.0	[30.0, 60.0]	32.5	[17.5, 47.5]	27.5	[15.0, 42.5]

Percentage of correct FCT results for each condition and repetition-interval pair with 95% CI in brackets (obtained through a bootstrap procedure using 5,000 samples).

Supplementary Table 4 | Correlation between measures in Experiment 1.

Measures	10 Presentations	5 Presentations	1 Presentation (Control Condition)
PAS and FCT	.79*	.90*	.06
PAS and CR	.91*	.96*	.28
FCT and CR	.93*	.93*	.63

Partial correlation between measures for each condition taking repetition intervals into account. Results show the partial relation coefficient *r*. Asterisks (*) indicate that results were significant with Bonferroni adjusted *p*-values < 0.01.

Supplementary Table 5 | Detailed Experiment 2 Results

Condition	35 ms Interval			70 ms Interval			139 ms Interval		
	PAS	CR	FCT	PAS	CR	FCT	PAS	CR	FCT
10 Presentations	1.65	80.8	84.2	0.97	55.0	67.5	0.69	34.2	55.0
1 Presentation (Control Cond.)	0.47	18.3	35.0	0.35	13.3	36.7	0.37	13.3	43.3

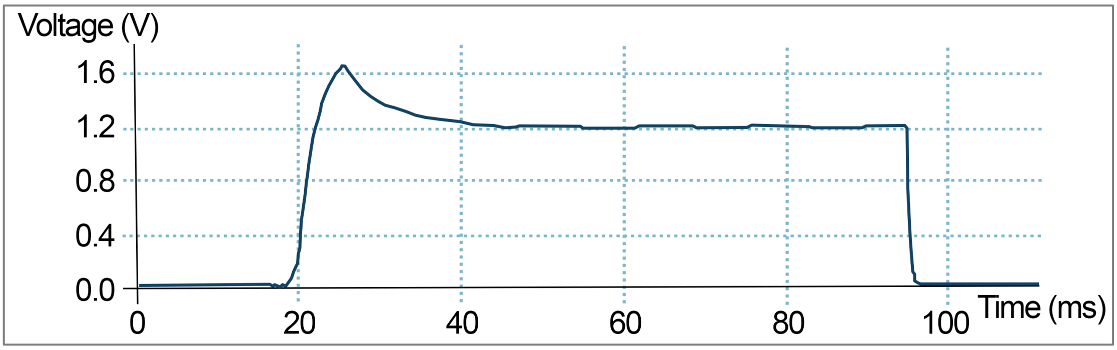

Supplementary Figure 3 | Display response time. Averaged results of 200 luminance measures of the display transition from black to grey and back to black based on photodiode and oscilloscope tests.

Supplementary Table 6 | Stimulus presentation timing.

Frames	Mean Display Time (ms)	95% CI (ms)
1	7.43	[7.39, 7.46]
2	14.37	[14.34, 14.41]
3	21.32	[21.28, 21.36]
4	28.27	[28.23, 28.31]
5	35.21	[35.17, 35.26]
6	42.16	[42.12, 42.21]
8	56.05	[56.01, 56.10]
10	69.95	[69.90, 70.00]
15	104.68	[104.62, 104.75]
20	139.42	[139.35, 139.49]
25	174.15	[174.07, 174.24]
30	208.89	[208.80, 208.98]
40	278.36	[278.25, 278.47]
60	417.30	[417.15, 417.45]
80	556.24	[556.05, 556.43]
100	695.18	[694.95, 695.41]
200	1389.88	[1389.46, 1390.31]
400	2779.29	[2778.48, 2780.10]
800	5558.10	[5556.52, 5559.69]
1200	8336.92	[8334.55, 8339.28]

Stimulus presentation timing based on commanded frames (display refresh cycles) and mean measures based on photodiode and oscilloscope tests with calculated confidence intervals. Nonlinearity is due to different combinations of rise frames (“painting” the screen), hold frames (maintaining the previous image), and fall frames (“erasing” the screen), which have unique temporal properties.
